# Effects of Pornography Use and Demographic Parameters on Sexual Response during Masturbation and Partnered Sex in Women

**DOI:** 10.3390/ijerph17093130

**Published:** 2020-04-30

**Authors:** Sean M. McNabney, Krisztina Hevesi, David L. Rowland

**Affiliations:** 1 Department of Psychology, Valparaiso University, Valparaiso, IN 46383, USA; sean.mcnabney@valpo.edu; 2 Department of Psychology and Education, Eötvös Loránd University, 1075 Budapest, Hungary; dr.hevesi.kriszta@gmail.com

**Keywords:** pornography, masturbation, partnered sex, sexual activity, sexual response, arousal, orgasm, orgasmic difficulty, orgasmic pleasure

## Abstract

The effect of pornography on sexual response is understudied, particularly among women. A multinational, community-based sample of 2433 women at least 18 years of age completed a 42-item, opt-in questionnaire collecting information on demographic and sexual history characteristics, use of pornography during masturbation, frequency of pornography use, and sexual response parameters. Pornography use and average frequency were compared across demographic variables. We also examined how pornography frequency predicted differences in self-reported arousal difficulty; orgasmic difficulty, latency, and pleasure; and the percent of sexual activities ending in orgasm during both masturbation and partnered sex. On average, women using pornography were younger, and reported more interest in sex. Pornography frequency differed significantly by menopausal status, sexual orientation, anxiety/depression status, number of sexual partners, and origin of data collection. During masturbation, more frequent pornography use predicted lower arousal difficulty and orgasmic difficulty, greater pleasure, and a higher percentage of masturbatory events leading to orgasm. Frequency of pornography use predicted only lower arousal difficulty and longer orgasmic latencies during partnered sex, having no effect on the other outcome variables. Pornography use frequency did not predict overall relationship satisfaction or sexual relationship satisfaction. Overall, more frequent pornography use was generally associated with more favorable sexual response outcomes during masturbation, while not affecting most partnered sex parameters. Several demographic and relationship covariates appear to more consistently and strongly predict orgasmic problems during partnered sexual activity than pornography use.

## 1. Introduction

With the advent of, and increasing access to, the internet and other private media forms such as DVD, traditional barriers to sexually explicit materials such as public stigma and societal restriction have weakened considerably, and the viewing of pornography has become commonplace [1,2]. In a comprehensive analysis of General Social Survey (GSS) data from 18,225 women in the United States, Wright and colleagues [3] observed that pornography use has remained largely consistent among US women ages 18–30 from the period 1973 to 2010, with approximately one in three viewing a pornographic movie within a year of completing the survey. Other sources of sexually explicit media have also been evaluated in recent years. For example, cross-cultural analysis of web traffic on PornHub (https://www.pornhub.com) has indicated that women users comprised about 29% of visitors to the site in the United States and Canada during 2018, with this percentage having increased in recent years [4]. 

Social restriction on access to pornography is predicated on the assumption that pornography imparts negative effects to individuals and thus to society at large. For years, these effects had been framed as moral or religious issues—ones involving temptation, sin, depravity, and the consequences of self-indulgence, promiscuity, and infidelity to the family unit [5,6]. More recently, these negative consequences have found secular parallels, often ones involving psychological health and social welfare. Such purported detrimental consequences include effects on an individual’s sexual response [7] and relationship satisfaction [8], the potential for addiction [9,10,11], objectification of (particularly) women [12,13], and encouragement of high-risk sexual behaviors [14,15]. On the other hand, pornography might also serve less sinister purposes, such as having an educational function for individuals having little understanding of sexual practices and behavior [16,17], or an erotic/arousal function for both men and women during masturbation [18]. Couples, too, may use pornography as a means of heightening both their sexual interest/desire and arousal during or preceding partnered sex [19]. Because our study focused on the relationship of pornography use in two major domains, orgasmic response and relationship satisfaction, we review these two outcomes in greater detail.

### 1.1. Pornography Use, Sexual Relationship Satisfaction, and Relationship Quality

A recent meta-analysis of 50 studies has provided evidence for a curvilinear association between frequency of pornography use and sexual relationship satisfaction, with detrimental effects to satisfaction occurring only after exceeding a particular threshold of use in men but not women [20]. Interestingly, no significant relationship was observed between pornography frequency and *intrapersonal* satisfaction, assessed by measures such as body satisfaction, self-esteem, and sexual self-esteem. A survey of 1513 young adults in the U.S. (nearly 62% women) also supported a curvilinear association between greater pornography viewing and decreased sexual satisfaction—this time for both men and women—but the acceleration of the curve was more pronounced for men [8]. Decrements to sexual relationship satisfaction were also more pronounced for individuals not currently in a romantic relationship and those identifying as religious. 

Not all studies have supported a straightforward interpretation between pornography use and sexual relationship satisfaction. In a convenience sample of 803 adults, cluster analyses revealed significant differences between participants using pornography primarily for recreation, those individuals emotionally or psychologically distressed about their pornography use, and compulsive users [21]. Recreational, non-compulsive users—with women comprising 78% of this cluster—reported greater sexual satisfaction and lower sexual avoidance compared to the other two profiles. Moreover, in a survey of 1291 adults in the United States, those who viewed pornography together with their partner reported significantly higher sexual satisfaction and greater relationship dedication than respondents who only watched pornographic content alone [22]. With regard to self-perception of potential benefits and problems arising from pornography consumption, Danish women and men attributed generally positive effects of pornography on sex life, attitudes toward sex, and life in general [23]. Interestingly, women reported slightly fewer negative effects of pornography use compared to their male counterparts.

### 1.2. Pornography Use, Sexual Education, and Pleasure

Under some circumstances, pornography use may contribute to sexual knowledge among women. Research carried out on a sample of 68 undergraduate heterosexual women revealed that exposure to pornographic films depicting concomitant clitoral stimulation throughout penile-vaginal, oral-penile, and hand-penile sexual behaviors increased participants’ inclusion of these techniques to their repertoire of activities during partnered sex [24], although it did not necessarily improve orgasmic consistency. This finding is supported by an earlier survey of 833 undergraduate students, in which more accurate conceptual knowledge of the clitoris was correlated with a higher frequency of orgasm during masturbation, but it was not associated with greater orgasmic frequency during partnered sex [25].

Researchers have also noted the potential for pornography to impart sexual techniques or behaviors that pose a risk to psychological health. For example, thematic content analysis of 50 commercially popular pornography films revealed that some films did not depict explicit verbal indicators of consent to sexual activity, and nonverbal indicators were not consistently salient [26]. In a similar vein, a survey of 140 heterosexual women in South Korea illustrated that the perception of pornography as a reliable source of sexual information was negatively correlated with the frequency of condom use during partnered sex [27], as was earlier initial exposure to pornography among Croatian young adults [28]. Recent reports have also supported a correlation between exposure to pornography depicting condom-less anal intercourse (“barebacking”) and unprotected anal sex between men [29,30], thus increasing the risk of HIV and infection. The prevalence of condom use for anal intercourse among male–female dyads also tends to be lower than that reported for penile–vaginal intercourse [31,32,33], even within the same individuals/couples, and this finding may be associated in part to depictions of condom-less anal intercourse in sexually explicit media. 

### 1.3. Pornography Use and Sexual Response/Orgasm in Women

Sexual response, pleasure, and orgasm in women are affected by many factors, one critically important factor being the type of sexual activity—whether masturbatory or with the partner. Specifically, the majority of women are able to masturbate to orgasm, but a significant portion (as high as 30%–40%) report orgasmic difficulty (OD) during partnered sex, especially when penile–vaginal intercourse constitutes the primary form of stimulation [34,35,36,37]. Furthermore, masturbatory activity per se has minimal enhancing effects on sexual pleasure and orgasmic response during partnered sex, unless the stimulatory activities during masturbation are transferred to activities during partnered sex [38]. One common type of stimulatory activity used primarily during masturbation, though much less frequently during partnered sex, is that of erotic/pornographic media. Specifically, pornography is often used by women to increase arousal and pleasure during masturbation, but its absence during partnered sex might deprive these women of an important and habitual source of sexual stimulation, thereby making it more difficult for them to become aroused and reach orgasm. 

### 1.4. Aims of the Current Study

In this study, we broadly addressed the issue of the relationship between pornography use and sexual response. Specifically, (1) we describe differences in pornography use based on a number of demographic, personal, and sexual response variables. We then asked how pornography use during masturbation might positively or negatively affect sexual/orgasmic responsivity (2) during masturbation and (3) during partnered sex, indicated by a number of outcome indices, including difficulty becoming aroused, difficulty reaching orgasm, orgasmic pleasure, orgasmic latency, and percent of time reaching orgasm. We further asked (4) whether pornography use during masturbation was related to the woman’s sexual and overall relationship satisfaction with her partner. As we assessed these relationships, we (5) controlled for the independent effects of a number of covariates known for affecting sexual response, including anxiety/depression [39,40,41], menopausal status [42], and a number of health and demographic factors, including ongoing medical issues, age [43], and education level [44,45]. 

Due to a primarily data-driven approach in this study, we did not formulate specific hypotheses a priori. Nevertheless, we approached our analyses with several expectations based upon prior literature, including assumptions that the frequency of pornography use would differ relative to particular demographic and sexual history variables, that pornography frequency use might predict women’s current sexual relationship satisfaction, and that pornography use would impact orgasmic outcomes related to masturbation differently than orgasmic outcomes during partnered sex.

## 2. Materials and Methods 

### 2.1. Participants

Participants for this study were drawn from a community-based sample of 2433 women ≥ 18 years of age (mean age = 28.72, SEM = 0.201, range = 18–95) in a current or recent (≤12 months ago) sexual relationship through three approaches. The first consisted of 966 women in the USA visiting one of 12 online postings on reddit.com, with promotion through social media (including Facebook postings), word-of-mouth, listservs, media announcements, and posted fliers. The second approach consisted of 1043 women visiting comparable online forums and promotions in Hungary or visiting the Hungarian research group’s webpage. The third approach consisted of 424 women enrolled in a professional degree program at a major university in Hungary who volunteered to take a pencil-and-paper version of the survey. Completion of the online version of the questionnaire occurred through self-selection, indicating a need for women ages 18+ to fill out a survey pertaining to sexual health/functioning. Participation in the pencil-and-paper survey was voluntary, as students in various courses could elect to use questionnaire completion as one of several options to fulfill a course requirement.

### 2.2. Survey Questionnaire

As part of the survey development, a pilot was conducted with three focus groups of women, one from the USA (*n* = 13, mean age = 27), and two from Hungary comprised of students from professional psychology (*n* = 13, mean age = 20) and students from non-professional disciplines (*n* = 9, mean age = 22). Groups reviewed survey items, commented on their relevance, suggested response categories, appraised overall item face-validity, ensured clarity of the items, and assessed the time required for survey completion [46]. For Hungarian participants, the survey was translated to Hungarian by a professional translator and then back-translated by a second translator to English to ensure preservation of meaning. 

The first seven questions of the 42-item, investigator-derived survey consisted of demographic information including age, menopausal status, level of education, and any ongoing/chronic medical conditions. The second portion examined factors relevant to respondents’ sexual histories over the past 9–12 months, such as self-rated importance of sex, number of current sexual partners, sexual orientation, sexual relationship satisfaction, overall relationship satisfaction, and frequency of partnered sexual activity ending in orgasm. This section also assessed masturbatory practices, including frequency of masturbation and use of pornography/erotic materials during masturbation. The third survey section evaluated parameters related to the sexual response cycle, including sexual desire, difficulty becoming aroused during masturbation and partnered sex, and insufficient production of vaginal lubrication. When applicable, questions were drawn from the Female Sexual Function Index (FSFI), which has been extensively validated for both clinical and non-clinical populations [47,48,49]. The final section of the survey assessed orgasmic response during both masturbation and partnered sex, examining factors such as type/methods of stimulation, derived pleasure, estimated latency to orgasm, difficulty reaching orgasm, and perceived reasons (attributions) for such difficulty during either masturbatory or partnered sexual activities. 

#### 2.2.1. Outcome Measures

Five sexual outcomes during partnered sex and masturbation (separately) were included for analysis: sexual arousal *difficulty*, orgasmic *difficulty*, likelihood of reaching orgasm, orgasmic latency, and orgasmic pleasure. Sexual arousal difficulty was assessed on a 5-point scale, with 1 = almost never, 5 = almost always. Likelihood of reaching orgasm was assessed on a continuous 100-point visual analog scale (1 = never, 100 = always). Orgasmic latency, assessed beginning with physical stimulation and the woman’s intention of trying to move toward orgasm, was measured on a 1–7 scale, with 1 = 1–5 min, 2 = 6–10 min, 3 = 11–15 min, 4 = 16–20 min, 5 = 21–30 min, 6 = ≥ 30 min, 7 = I do not reach orgasm [50]. Orgasmic pleasure was measured on a 0–5 scale, with 0 = I do not reach orgasm during partnered sex, 1 = not pleasurable or satisfying, 5 = very pleasurable or satisfying [51]. Orgasmic difficulty was measured on a 1–6 scale, with 1 = almost never, 5 = almost always, 6 = I do not reach orgasm [52,53]. For all measures, respondents were asked to use their current or most recent sexual partner as their reference, or the past 6–12 month timeframe (e.g., for masturbation). In addition to these outcomes, we assessed respondents’ overall relationship satisfaction and sexual relationship satisfaction (e.g., “How satisfied are you with your primary *sexual* relationship, that is, with the sexual relationship you consider to be most significant to you?”) during the previous 12 months, both of which were measured using 5-point scales where 1 = not at all satisfied, 5 = very satisfied. The precise wording for each of the above items is included in Appendix A: Supplementary Information on the Survey Questionnaire (Table A1, Table A2).

#### 2.2.2. Predictor Covariates

Pornography use—its presence, absence, and frequency—along with scaling and/or categories for all other predictor covariates (including origin-of-data, whether online (Hungary or USA), or onsite), are described in detail in Table 1. For some variables, category frequencies were low (e.g., non-heterosexual orientations), thus resulting in combining categories.

### 2.3. Procedure

The final version of the anonymous survey took approximately 20 min to complete. Project approval was obtained from the Institutional Review Boards at the investigators’ universities in both Hungary and the United States. Informed consent was given by participants, with their needing to check boxes attesting (i) to their being ≥ 18 years old, and (ii) to their informed consent prior to accessing the survey. Respondents were notified that they could end participation in the study at any point by closing the questionnaire (no data were saved until final submission of the survey).

### 2.4. Design and Analytic Strategy

Preliminary comparisons across demographic and sexual response variables were made with *t* or comparable tests, with α = 0.01 due to the number of comparisons. Then, eight separate ordinal regression analyses and two logistic quasi-binomial regressions for orgasm percent (see [54]) were carried out to understand how pornography use, along with several key demographic, lifestyle, and sexual history variables, accounted for variance in five outcome variables related to sexual response and performance: arousal difficulty, orgasmic difficulty, orgasmic latency, orgasmic pleasure, and percentage of time reaching orgasm. These outcome measures were assessed separately for masturbation and partnered sexual activity, hence five analyses for masturbation and five for partnered sex. Our goal in running this number of multiple regression analyses was to determine whether pornography use was broadly and consistently associated with sexual response parameters in women *by discerning patterns of repeating significant covariates,* and for this reason we retained α at 0.05 for these analyses. Two final ordinal regressions were carried out using sexual relationship satisfaction and overall relationship satisfaction as outcome variables. 

#### Assessing Collinearity through Bivariate Correlations

As a preliminary step, to control for collinearity among the outcome variables and between predictor covariates, Spearman rank-order correlations were evaluated, with removal of one of the pair of variables showing correlations ≥ 0.60 [55], unless otherwise noted. While moderate associations were observed among the outcome variables, the only pair of variables meeting the 0.60 threshold for collinearity was orgasmic difficulty and the percentage of time reaching orgasm during partnered sex (r_s_ = 0.731, *p* < 0.01). We ultimately decided to retain regression analyses for both outcome variables, despite their relatively strong association. In contrast, none of the associations between predictor covariates reached the 0.60 threshold for collinearity. Thus, all the predictor covariates were retained in the regression analyses.

## 3. Results

### 3.1. Aim 1: Sample Subgroups, Pornography Use, and Differences between Users and Non-Users

Characteristics and group sizes for all predictor covariates are presented in Table 1. Participants were typically well-educated (27.6% completing a college bachelor’s degree and 28.3% acquiring postsecondary technical instruction/skill certification), premenopausal (92%), and currently involved in a sexual relationship (81.1% had one or more sexual partners). 

Pornography use during masturbation was higher in specific subgroups, including premenopausal (vs. peri- or postmenopausal) women; women reporting persistent anxiety or depression over the past 6–12 months; non-heterosexual women; women having two or more partners; and US women (vs. Hungarian women). 

Sexual history and response variables comparing masturbation and partnered sex activities are presented in Table 2. Women who use pornography during masturbation tend to be younger, indicate a higher interest in sex, masturbate more frequently, have lower arousal difficulty during masturbation, are more likely to reach orgasm during masturbation, and have longer latencies during masturbation. They also have greater difficulty reaching orgasm during partnered sex and greater distress regarding orgasmic difficulty during partnered sex. 

### 3.2. Aim 2: Relationship of Use of Pornography to Masturbation Outcome Variables

Table 3 depicts the results of the five regression analyses for the masturbation outcome variables, with use of pornography significantly related to all five. Specifically, during masturbation, *more* frequent use of pornography was related to less difficulty becoming aroused, less orgasmic difficulty, longer orgasmic latencies, greater orgasmic pleasure, and higher percent of time reaching orgasm. 

### 3.3. Aim 3: Relationship of Use of Pornography during Masturbation to Partnered Sex Outcome Variables

Table 4 depicts the results of the five regression analyses for the partnered sex outcome variables, with use of pornography significantly related to only two of the five, namely arousal difficulty and orgasmic latency. Specifically, those women who used pornography *more frequently* also reported less difficulty becoming sexually aroused and longer latencies during partnered sex. 

### 3.4. Aim 4: Pornography Use and Sexual and Overall Relationship Satisfaction

Table 5 shows the relationship between pornography use during masturbation and both sexual relationships satisfaction and overall relationship satisfaction (for participants’ stated primary relationship). Frequency of pornography use was not related to either partner–relationship variable.

### 3.5. Aim 5: Role of Demographic and Personal Covariates

Several other covariates were *consistently* related to the sexual response outcome variables during masturbation and partnered sex. 

*Education level*. Most consistent was participants’ education level, which was significantly related to three of five outcomes during masturbation, and four of five outcomes during partnered sex. Specifically, during masturbation, lower education levels were associated with greater arousal difficulty, greater orgasmic difficulty, and longer latencies to orgasm. During partnered sex, lower education levels were associated with greater orgasmic difficulty, longer latencies to orgasm, lower orgasmic pleasure, and lower percent of times reaching orgasm. Education level was, however, not related to either sexual or overall relationship satisfaction.

*Sexual relationship satisfaction* predicted arousal difficulty and orgasmic difficulty during masturbation, and strongly affected all sexual response parameters during partnered sex. Specifically, greater sexual relationship satisfaction was associated with lower arousal and orgasmic difficulties during masturbation, and lower arousal and orgasmic difficulties, shorter latencies to orgasm, greater orgasmic pleasure, and greater percent of time reaching orgasm during partnered sex. 

*Self-reported anxiety and depression* was related to greater orgasmic difficulties and percent of times reaching orgasm during masturbation, and greater arousal difficulty during partnered sex. Anxiety and depression were both associated with lower sexual and overall relationship satisfaction.

*Number of sexual partners* was related only to shorter orgasmic latency during masturbation, and was related to three of five sexual outcome measures during partnered sex. Specifically, having more sexual partners was associated with greater arousal difficulty, greater orgasmic difficulty, and lower orgasmic pleasure. 

Other regression covariates showed substantially less consistency or were sporadic in predicting sexual outcome variables during masturbation and/or partnered sex. For example, being peri- or post-menopausal was associated with greater arousal difficulty during masturbation, and greater orgasmic difficulty, longer orgasmic latencies, and lower orgasmic pleasure during partnered sex. Having an ongoing medical issue was minimally related to the outcome measures, being associated only with lower percent of time reaching orgasm during partnered sex. Sexual orientation was not associated with any of the masturbation or partnered sex outcomes. 

### 3.6. Origin of Data

All regression analyses controlled for the origin of data, whether from U.S. vs. Hungarian women. In doing so, the various effects of pornography use occurred despite differences in the origin of the data. However, U.S. and Hungarian women differed in several ways worth mentioning. In general, U.S. women reported more difficulties with, and lower positive outcomes regarding, orgasmic response during masturbation. US women also reported greater orgasmic difficulty during partnered sex as well as lower relationship satisfaction.

## 4. Discussion

Research pertaining to pornography has traditionally been conducted from three perspectives. First, pornography use has been evaluated in relation to couples’ relationship satisfaction, often under the assumption that greater reliance upon erotic materials during masturbation is associated with poorer relationship outcomes [8,20,22]. Second, pornography has been studied from a neurobehavioral perspective to determine whether individuals who report “compulsive” use exhibit neurophysiologic or epigenetic alterations that typify models of addiction [11,56,57]. Third, content analyses of pornographic materials have been conducted to examine the extent to which exposure to beneficial/educational (e.g., providing clitoral stimulation during partnered sex), risky (e.g., condom-less sexual activity), or demeaning (e.g., lack of verbal consent during intercourse, sexual aggression, etc.) sexual scripts might increase the preponderance or social acceptability of such behaviors [24,26,27,58,59]. Despite the insights gained from these approaches, few studies have analyzed putative direct effects of pornography use on the sexual response cycle, including arousal and orgasmic parameters, comparing masturbatory and partnered sexual activities. 

In stark contrast to prior research findings and public opinion, we did *not* find strong empirical support for the hypothesis that pornography use is consistently associated with greater sexual dysfunction or relationship dissatisfaction. In all five regression analyses related to masturbation, more frequent pornography use predicted *greater* ease of becoming aroused and reaching orgasm, longer latencies to orgasm, greater pleasure upon orgasm, and a higher percentage of masturbatory events leading to orgasm. Among the same parameters for partnered sexual activity, more frequent pornography use predicted greater ease becoming aroused and longer latencies, but no significant associations, either positive or negative, were observed for any of the other outcomes in the regression analyses. Overall, women’s use of pornography to enhance orgasmic response and pleasure was strongly supported by our findings, yet pornography use during masturbation appeared to have no deleterious effects on sexual functioning during partnered sex. Indeed, it was actually associated with lower arousal difficulty during partnered sex. Furthermore, *no associations* were observed between pornography use frequency and general relationship satisfaction or sexual relationship satisfaction with one’s primary partner in the previous 12 months. In these respects, our results highlight the potential positive effects that pornography use might have for women’s enjoyment of sex. Furthermore, to the extent that pornography use might represent greater openness to using less conventional strategies for enhancing their sexual experience, it may reflect one of a number of techniques that differentiate women who use pornography from women who do not [38,60].

As a therapeutic consideration, women who viewed pornography reported significantly greater distress when they were unable to reach orgasm during partnered sexual activity. Moreover, there was a trend (*p* = 0.062) for these women to more strongly endorse the belief that their sexual partners were distressed by their orgasmic difficulty compared to participants who did not report pornography use. Several possibilities might account for these findings. One interpretation relies upon the observation that the vast majority of mass-produced pornographic content devalues the contours of a romantic relationship—including foreplay and a gradual buildup to sexual activity—and emphasizes the sexual act itself, with orgasm often portrayed most vividly [18,61,62]. In this view, repeated exposure to erotic materials might shift a woman’s experience of sexual pleasure from a nonlinear model emphasizing intimacy and sexual satisfaction [63,64,65] toward a more goal-directed, orgasm-centric perspective. Alternatively, since respondents who indicated using pornography also indicated greater interest in sex, failure to reach orgasm may elicit stronger disappointment for them than for individuals who perceive sex as less important or captivating. Experimental research designs examining OD-related distress when exposed to different subgenres of pornographic content (e.g., between mainstream and feminist materials [62]) might help to clarify the direction and scope of these associations. 

While frequency of pornography use did not consistently predict orgasmic problems, several of the covariates in our regression models were associated with disruption of sexual functioning. For example, ongoing anxiety/depression was associated with difficulty reaching orgasm and a lower percent of time reaching orgasm during masturbation, and greater difficulty becoming aroused during partnered sexual activity. Moreover, greater anxiety/depression predicted lower relationship satisfaction and sexual satisfaction. These findings align with prior research demonstrating that anxiety and depression may increase the individual’s cognitive distraction or negative affect [66,67], both of which can shift attention away from sexual behaviors or partner cues and onto competing, non-erotic stimuli [41,68,69,70]. 

Education also emerged as a consistent predictor in our regression models, as higher levels of educational attainment were associated with less arousal difficulty during masturbation; greater orgasmic pleasure and a higher likelihood of orgasm during partnered sex; and less difficulty reaching orgasm and shorter latencies to orgasm across both masturbation and partnered sex. These findings are consonant with prior reports where educational attainment was associated with greater orgasmic functioning and a lower likelihood of experiencing sexual problems [71,72], even when controlling for other demographic variables such as underlying medical issues. One common interpretation of this relationship is that more educated (and thus literate in many cultures) women are more aware of their sexuality, sexual issues, and sexual choices, and thus able to better articulate their sexual desires to their partner and to prioritize their own pleasure as an integral component of meaningful sexual relationships [73]. Yet, while such relationships within emerging nations may not be surprising, the fact that similar relationships are found in highly literate societies as well is notable. 

Finally, we observed a relatively consistent effect from the data collection site, as recruitment from Hungary was associated with a greater likelihood of reaching orgasm during partnered sex, as well as less difficulty becoming aroused, shorter latencies, and greater pleasure during masturbation. Somewhat surprisingly, these predictions occurred in the regression models despite an association between the collection sites in Hungary and lower general relationship satisfaction relative to respondents in the USA. Ongoing work in this field that incorporates multinational sampling can help determine the extent to which cross-cultural differences in pornography use and attitudes toward masturbation translate into meaningful differences during partnered sexual activity [4]. 

### Strengths and Limitations

Our study included a relatively robust sample size with wide distribution, a common benefit of online/non-online survey data [46]. Moreover, the cross-cultural nature of our study sample—with respondents from both the United States and Hungary—allowed us to compare the frequency of pornography use between two Westernized countries. Since topics related to sexual identity and behavior are often perceived as sensitive, the anonymity of an online approach, including participants’ ability to complete the questionnaire in a private location of their choosing, may have facilitated more candid responses and reduced issues pertaining to social desirability [74]. Finally, by designing two survey questions to measure pornography use as both a dichotomous and a scaled variable, we were better equipped to capture the complex relationships between pornography, the empirically derived covariates, and sexual response outcomes.

Despite the strengths of this research design, we also acknowledge its restrictions and potential limitations. First, the cross-sectional nature of the study generally precluded establishing the direction of associations between pornography use and sexual function. While more frequent pornography use predicted greater ease reaching orgasm during masturbation and higher arousal during partnered sexual activity, for example, we cannot determine whether respondents are using pornography to achieve these desired outcomes or, alternatively, whether participants who report greater sexual functioning are more motivated to seek out arousing stimuli—including pornographic media. Longitudinal study designs could also demonstrate whether the frequency of pornography use tends to remain consistent across the life course or when fluctuations most commonly occur. 

Another limitation is the inability of our questionnaire to distinguish between multiple reasons for pornography use, such as to enhance/augment pleasure (approach motivation) or to compensate for decreased intimacy with one’s partner (avoidance motivation). Similar to prior reports on masturbation [36] and partnered sex [75,76], one might reasonably hypothesize that underlying motivations for and attitudes toward pornography use could differentially affect sexual response. Finally, our questionnaire did not distinguish between women who were satisfied with their pornography use and those who either felt distressed about using pornography or addicted to such materials (e.g., [21]). Multiple reports have demonstrated that the *perception* of pornography addiction or compulsivity, rather than pornography use frequency itself, may be a stronger predictor of sexual dysfunction among both men and women [77,78].

## 5. Conclusions

In our multinational, cross-sectional survey of pornography use and sexual response in women, higher frequency of pornography use predicted greater sexual functioning across all outcome variables during masturbation, yet had no deleterious effects on sexual outcomes during partnered activity. Across masturbation and partnered sex, lower levels of educational attainment and the presence of ongoing anxiety or depression were the two most consistent predictors of orgasmic dysfunction in the regression models. Greater sexual relationship satisfaction, in contrast, was associated with more favorable outcomes during partnered sex. Taken together, these findings suggest that the frequency of pornography use per se does not contribute to sexual problems during partnered sex., However, it could well be that a subset of women use erotic materials to compensate for psychosocial factors (e.g., dissatisfaction in one’s sexual relationships, ongoing anxiety or depression) that independently impinge upon sexual responsivity. 

## Figures and Tables

**Table 1:** Demographic characteristics for participants and their relationship to pornography use.

Variables	*N* (%)	Comparison of Pornography Use ^1^			
Demographic Variable		Mean (SEM)	Test Statistic ^2^	*p*-Value	Effect Size ^3^
**Level of Education**					
High school or equivalent	201 (8.3%)	2.87 (0.125)	r = 0.035	0.106	N/A
Skill certification/technical degree	686 (28.3%)	2.56 (0.062)			
Some college	433 (17.9%)	3.31 (0.080)			
Undergraduate (bachelor’s) degree	669 (27.6%)	2.87 (0.064)			
Graduate/post-baccalaureate study	435 (17.9%)	2.80 (0.077)			
**Menopausal Status**					
Pre-menopausal	2174 (92%)	2.88 (0.036)	t (2118) = 3.590	<0.001	0.156
Peri- or post-menopausal	189 (8%)	2.44 (0.116)			
**Current/Ongoing Medical Issues**					
No	1965 (83.1%)	2.83 (0.037)	t (2176) = −0.647	0.518	−0.028
Yes	398 (16.8%)	2.89 (0.080)			
**Persistent Anxiety/Depression**					
No	1841 (77.8%)	2.74 (0.038)	t (2176) = −5.799	<0.001	−0.249
Yes	524 (22.1%)	3.22 (0.073)			
**Sexual Orientation**					
Heterosexual	1948 (85.4%)	2.70 (0.036)	t (475.516) = −11.087	< 0.001	−1.017
Non-heterosexual	334 (14.6%)	3.66 (0.078)			
**Current Sexual Partner**					
No current sexual partner	431 (18.9%)	2.91 (0.083) ^A,B^	F_W_ (2304.285) = 5.851	0.003	0.006
1 partner	1717 (75.3%)	2.79 (0.038) ^B^			
2 or more partners	133 (5.8%)	3.29 (0.148) ^A^			
**Origin of Data Collection**					
Online survey, United States	966 (39.7%)	3.61 (0.050) ^A^	F_W_ (2959.943) = 178.799	<0.001	0.136
Online survey, Hungary	1043 (42.9%)	2.34 (0.046) ^B^			
Pencil-and-paper questionnaire, Hungary	424 (17.4%)	2.63 (0.081) ^C^			
**Pornography Use during Masturbation**					
I do not masturbate	132 (5.9%)	---			
No	802 (35.8%)	1.71 (0.042)	t (1805.470) = −34.904	<0.001	−1.490
Yes	1307 (58.3%)	3.64 (0.036)			
**Frequency of Pornography Use**					
I do not use erotic materials	751 (34.5%)	---	---	---	---
Only several times each year	185 (8.5%)				
Less than once a month	332 (15.2%)				
Several times a month	508 (23.3%)				
Several times per week	368 (16.9%)				
Multiple times per day	34 (1.6%)				

The names of the demographic variables are represented with bold font. ^1^ Evaluated as frequency of pornography use, where 0 = I do not use erotic materials, 1 = only several times a year, 2 = less than once a month, 3 = several times a month, 4 = several times a week, 5 = several times a day. ^2^ For demographic variables with two response categories, independent-sample *t*-tests were computed. For demographic variables with more than two response categories, one-way Welch’s ANOVA tests (F_W_) were computed with Games–Howell post-hoc analyses. Significant differences (α = 0.01) between subgroups are denoted by different superscripts (A,B,C). ^3^ Effect sizes are reported as Cohen’s d values (for independent-sample *t*-tests) or partial η^2^ values (for ANOVAs). Interpretation using absolute values for Cohen’s d are as follows: 0.2 (small effect), 0.5 (medium/intermediate effect), 0.8 (large effect). Interpretation of effect sizes using partial η^2^ values are as follows: 0.01 (small effect), 0.06 (medium/intermediate effect), 0.14 (large effect).

**Table 2:** Comparison of women’s sexual response outcomes based upon whether or not they engage in pornography use during masturbation.

Variables	Yes		No		*p*-Value ^1^	Effect Size ^2^
Sexual History and Response Outcomes	*N*	Mean (SEM)	*N*	Mean (SEM)		
**Sexual History Variables**						
Age	1303	27.76 (0.242)	793	30.64 (0.394)	<0.001	0.335
Importance of Sex	1306	4.01 (0.027)	797	3.91 (0.036)	0.028	−0.110
Interest in Sex	1300	4.27 (0.024)	800	4.04 (0.034)	<0.001	−0.245
Overall Relationship Satisfaction	1078	4.07 (0.029)	663	4.09 (0.037)	0.619	0.024
Sexual Relationship Satisfaction	1081	3.87 (0.033)	668	3.95 (0.041)	0.114	0.076
**Masturbation Outcomes**						
Frequency of Masturbation	1305	4.83 (0.051)	799	3.81 (0.069)	<0.001	−0.589
Percent Masturbation Ending in Orgasm	1299	90.54 (0.627)	791	86.66 (0.925)	0.001	−0.180
Arousal Difficulty	1289	1.73 (0.028)	764	1.87 (0.040)	0.005	0.147
Orgasmic Difficulty (OD)	1291	1.56 (0.029)	763	1.66 (0.040)	0.031	0.111
Orgasmic Latency (OL)	1265	2.27 (0.041)	751	2.08 (0.053)	0.004	−0.145
Orgasmic Pleasure (OP)	1264	3.76 (0.033)	754	3.74 (0.044)	0.676	−0.021
Self-Distress Regarding Masturbatory OD	1258	1.18 (0.046)	747	1.04 (0.053)	0.041	−0.098
**Partnered Sex Outcomes**						
Frequency of Partnered Sex	1242	6.47 (0.044)	768	6.43 (0.057)	0.511	0.001
Percent Partnered Sex Ending in Orgasm	1240	62.67 (0.945)	769	66.42 (1.129)	0.011	0.123
Arousal Difficulty	1227	1.79 (0.030)	750	1.78 (0.039)	0.798	−0.011
Orgasmic Difficulty (OD)	1211	2.85 (0.046)	757	2.66 (0.057)	0.009	−0.118
Orgasmic Latency (OL)	1222	3.72 (0.052)	766	3.52 (0.067)	0.016	−0.108
Orgasmic Pleasure (OP)	1193	3.81 (0.048)	746	3.90 (0.058)	0.231	0.059
Self-Distress Regarding Partnered Sex OD	1192	3.47 (0.045)	745	3.14 (0.055)	<0.001	−0.230
Partner Distress Regarding Partnered Sex OD	1185	3.63 (0.045)	742	3.49 (0.060)	0.062	−0.096

^1^ To compare the effect of pornography disuse/use on sexual history and outcome variables, independent-sample *t*-tests were computed, using α = 0.01 due to the number of comparisons. ^2^ Effect sizes are reported as absolute values of Cohen’s d and are as follows: 0.2 (small effect), 0.5 (medium/intermediate effect), 0.8 (large effect).

**Table 3:** Ordered logistic regression results for predicting arousal difficulty and orgasmic difficulty, latency, and pleasure during masturbation. Fractional response regression results for predicting the percent of time reaching orgasm during masturbation.

Regression Covariates	Arousal Difficulty	Orgasmic Difficulty	Orgasmic Latency	Orgasmic Pleasure	Orgasm Percent
Predictor	Coefficient (SE)	Coefficient (SE)	Coefficient (SE)	Coefficient (SE)	Coefficient (SE)
Level of Education	−0.133 (0.036) ***	−0.120 (0.039) **	−0.113 (0.035) **	0.058 (0.034)	0.461 (0.261)
Menopausal Status	0.419 (0.173) *	0.086 (0.183)	0.218 (0.159)	−0.288 (0.157)	0.202 (0.248)
Ongoing Medical Issues	0.045 (0.116)	−0.184 (0.124)	−0.057 (0.112)	−0.149 (0.111)	0.012 (0.165)
Ongoing Anxiety or Depression	−0.183 (0.110)	−0.337 (0.117) **	−0.201 (0.108)	0.140 (0.107)	−0.395 (0.153) **
Sexual Orientation	0.027 (0.128)	−0.111 (0.137)	−0.241 (0.124)	0.040 (0.124)	0.192 (0.191)
Sexual Relationship Satisfaction	−0.080 (0.033) *	−0.078 (0.036) *	−0.002 (0.032)	0.001 (0.032)	0.047 (0.057)
Number of Sexual Partners	0.027 (0.123)	−0.005 (0.134)	−0.278 (0.119) *	0.084 (0.118)	−0.177 (0.209)
Frequency of Pornography Use	−0.174 (0.030) ***	−0.099 (0.032) **	0.101 (0.029) ***	0.146 (0.028) ***	0.180 (0.044) ***
Origin of Data Collection ^1^	0.475 (0.106) ***	0.225 (0.116)	0.216 (0.102) *	−0.808 (0.102) ***	0.377 (0.161) *

Note: Significant predictors indicated by: * *p* < 0.05, ** *p* < 0.01, *** *p* < 0.001. ^1^ For these regression analyses, the two Hungarian samples (online and onsite) have been combined into a single group for comparison with the online USA sample.

**Table 4:** Ordered logistic regression results for predicting arousal difficulty and orgasmic difficulty, latency, and pleasure during partnered sexual activity. Fractional response regression results for predicting the percent of time reaching orgasm during partnered sexual activity.

Regression Covariates	Arousal Difficulty	Orgasmic Difficulty	Orgasmic Latency	Orgasmic Pleasure	Orgasm Percent
Predictor	Coefficient (SE)	Coefficient (SE)	Coefficient (SE)	Coefficient (SE)	Coefficient (SE)
Level of Education	0.017 (0.036)	−0.149 (0.034) ***	−0.130 (0.033) ***	0.095 (0.036) **	0.631 (0.144) ***
Menopausal Status	−0.069 (0.161)	0.466 (0.151) **	0.361 (0.148) *	−0.465 (0.169) **	0.217 (0.130)
Ongoing Medical Issues	−0.038 (0.118)	0.099 (0.109)	0.064 (0.109)	−0.073 (0.119)	0.233 (0.092) *
Ongoing Anxiety or Depression	−0.371 (0.112) **	−0.213 (0.106)	−0.208 (0.107)	0.177 (0.114)	−0.127 (0.088)
Sexual Orientation	−0.041 (0.134)	0.169 (0.124)	−0.010 (0.125)	−0.048 (0.135)	0.025 (0.102)
Sexual Relationship Satisfaction	−0.388 (0.034) ***	−0.427 (0.032) ***	−0.310 (0.032) ***	0.581 (0.036) ***	0.505 (0.032) ***
Number of Sexual Partners	0.793 (0.123) ***	0.496 (0.117) ***	0.228 (0.118)	−0.788 (0.127) ***	0.224 (0.119)
Frequency of Pornography Use	−0.092 (0.030) **	0.004 (0.028)	0.056 (0.028) *	0.047 (0.030)	0.026 (0.024)
Origin of Data Collection ^1^	−0.014 (0.109)	0.299 (0.101) **	−0.031 (0.102)	−0.317 (0.111)	0.417 (0.088) ***

Note: Significant predictors indicated by: * *p* < 0.05, ** *p* < 0.01, *** *p* < 0.001. ^1^ For these regression analyses, the two Hungarian samples (online and onsite) have been combined into a single group for comparison with the online USA sample.

**Table 5:** Ordered logistic regression results for predicting overall relationship satisfaction and sexual relationship satisfaction.

**Regression Covariates**	**Relationship Satisfaction**	**Sexual Satisfaction**
Predictor	Coefficient (SE)	Coefficient (SE)
Level of Education	0.012 (0.037)	−0.025 (0.036)
Menopausal Status	−0.044 (0.164)	−0.131 (0.162)
Ongoing Medical Issues	−0.046 (0.119)	0.264 (0.116) *
Ongoing Anxiety or Depression	0.588 (0.116) ***	0.507 (0.114) ***
Sexual Orientation	−0.086 (0.135)	0.125 (0.132)
Number of Sexual Partners	−0.118 (0.148)	0.122 (0.151)
Frequency of Pornography Use	−0.046 (0.031)	0.015 (0.030)
Origin of Data Collection ^1^	0.556 (0.110) ***	−0.014 (0.107)

Note: Significant predictors indicated by: * *p* < 0.05, *** *p* < 0.001. ^1^ For these regression analyses, the two Hungarian samples (online and onsite) have been combined into a single group for comparison with the online USA sample.

## Appendix A. Supplementary Information on the Survey Questionnaire

Below we have provided additional information on the questionnaire items relevant to our regression analyses, including the empirically derived covariates (Table A1) and the outcome variables of interest (Table A2).

**Table A1:** Survey questions for the regression covariates.

Variable Name	Survey Question	Values/Levels
Level of Education	What is your highest level of education completed?	1 = High school 2 = Skill/technical certification 3 = Some college 4 = Undergraduate degree 5 = Graduate/post-bac. study
Menopausal Status	Are you currently…?	1 = Before menopause 2 = Going through menopause 3 = After menopause 4 = N/A (e.g., ovaries removed)
Medical Issues	Do you have ongoing medical issues for which you are being treated?	0 = No 1 = Yes
Anxiety/Depression	Are you currently suffering from ongoing/persistent (> 6 months) anxiety or depression?	0 = No 1 = Yes
Sexual Orientation	What is your sexual orientation?	1 = Heterosexual 2 = Homosexual 3 = Bisexual 4 = Asexual 5 = Other (please describe)
Sexual Relationship Satisfaction	How satisfied are you with your primary *sexual* relationship, that is, with the sexual relationship you consider to be most significant to you?	0 = I do not have a sexual relationship at this time 1–5 scale; higher numbers correspond to greater satisfaction
Number of Sex Partners	Do you currently have a sexual partner?	0 = I do not have a sexual partner 1 = I have one sexual partner 2 = I have two or more sexual partners
Frequency of Pornography Use	About how often do you use erotic materials (e.g., print, video, Internet, audio)?	0 = Never 1 = Only several times a year 2 = Less than once a month 3 = Several times a month 4 = Several times a week 5 = Multiple times per day

**Table A2:** Survey questions for the outcome variables.

Variable Name	Survey Question(s) ^1^	Values/Levels
Arousal Difficulty	Do you have difficulty becoming or feeling sexually aroused during masturbation ^2^/sex with your partner?	0 = I do not masturbate/have sex 1–5 scale; higher numbers correspond to greater difficulty
Orgasmic Difficulty	If you masturbate/when you have sex with your partner, do you ever have problems reaching orgasm?	0 = I do not masturbate/have sex 1–5 scale; higher numbers correspond to greater difficulty
Orgasmic Latency	If you masturbate/have sex with your partner and are able to reach orgasm, about how long does it take for you, on average, from the time that you begin genital stimulation?	0 = I do not masturbate/have sex 1 = 1–5 min 2 = 6–10 min 3 = 11–15 min 4 = 16–20 min 5 = 21–30 min 6 = Longer than 30 min 7 = I do not reach orgasm
Orgasmic Pleasure	If you masturbate/have sex with a partner, how pleasurable or satisfying would you rate your typical orgasm?	0 = I do not reach orgasm 1–5 scale; higher numbers correspond to greater pleasure
Orgasm Percent	Estimate how often sexual activity with your partner/masturbation ends in orgasm for you by placing an X on the line below. If you have not had any sexual activities with a partner/do not masturbate, select N/A (not applicable).	0–100% (visual analog scale)
Overall Relationship Satisfaction	Beyond sexual issues, how satisfied overall are you with your primary relationship, that is, with the relationship you consider to be most significant to you?	0 = I do not have a relationship at this time 1–5 scale; higher numbers correspond to greater satisfaction
Sexual Relationship Satisfaction	How satisfied are you with your primary sexual relationship, that is, with the sexual relationship you consider to be most significant to you?	0 = I do not have a sexual relationship at this time 1–5 scale; higher numbers correspond to greater satisfaction

^1^ The questionnaire provides separate items for orgasmic parameters during masturbation and partnered sexual activity, but they are summarized together in this table due to their analogous coding. ^2^ In all relevant questions, masturbation is defined in the context of being “alone, without your partner present” to distinguish it as a solitary activity (i.e., not mutual masturbation, etc.).
